# A South African university-practitioner partnership to strengthen capacity in social and behaviour change communication

**DOI:** 10.3402/gha.v6i0.19300

**Published:** 2013-01-24

**Authors:** Nicola J. Christofides, Sara Nieuwoudt, Shereen Usdin, Susan Goldstein, Sharon Fonn

**Affiliations:** 1 School of Public Health, Faculty of Health Sciences, University of the Witwatersrand, Johannesburg, South Africa; 2 Soul City Institute for Health and Development Communication, Johannesburg, South Africa

**Keywords:** SBCC, competency-based curriculum, south–south collaboration, institutional capacity strengthening, Africa, practitioner academic partnership, donor responsiveness

## Abstract

Globally, communication plays an integral role in public health strategies, from infectious diseases to diseases related to lifestyles. The evolution of the field of social and behaviour change communication (SBCC), combined with the need for evidence based practice and multi-level interventions to promote health, and human resource gaps in sub-Saharan Africa have led to the imperative to standardise and formalise the field. Moreover, current practitioners come from different disciplinary backgrounds underlining the need to define common core skills and competencies. This paper describes the partnership between the Wits School of Public Health and the Soul City Institute for Health and Development Communication and how the partners responded to this need. It highlights the factors influencing sustainable institutional capacity to provide quality assured, accredited training. We describe an unexpected positive response from a number of practitioner organisations that have chosen to send multiple staff members for training, specifically to build a critical mass within their organisations. Finally, we note the interest from (mostly) southern-based academic institutions in setting up similar programmes and postulate that south–south collaborations can contribute to building sustainable context specific and evidence-informed SBCC programmes in the global south.

Significant resources have been invested to eradicate disease and decrease preventable mortality and morbidity, from communicable and non-communicable causes. These investments have acknowledged that individuals need to act differently to achieve health improvements. Even biomedical interventions, such as voluntary medical male circumcision, invest in communication to drive demand for clinic-based services (1, 2).

Individual change, while important, only impacts on the health profile of a country when there is a significant shift in collective behaviour towards more healthful activities (3). Public health is concerned primarily with this population-level change. Under the umbrella of public health, social and behaviour change communication (SBCC) develops, implements, and evaluates appropriate, evidence-based interventions to change population health.

SBCC has its origins in, and draws from, a range of academic disciplines including media, communication, and public health. There have been parallel and overlapping approaches that have emerged from these disciplines including communication for development, health communication, and health promotion (Fig. 1). SBCC does not replace any of these fields but draws from all of them. The Ottawa and Bangkok charters on health promotion, outlined changing health outcomes through advocating for supportive policies and supportive environments, community action, preventive and promotive health services, and increased health literacy (4, 5). Health communication programmes *are based on ‘*the scientific development, strategic dissemination, and critical evaluation of relevant, accurate, accessible, and understandable health information communicated to and from intended audiences to advance the health of the public’ (p. 2051) (6). Communication for development similarly addressed the need to support two-way communication systems but emphasised dialogue and the participation of communities in the decisions related to their development. It also sought change at different levels including the development of policies that promote development (7). In the late 1990s, the Rockefeller Foundation, in collaboration with a number of organisations from the global south, began looking at communication for social change, moving away from the top down, individual approach to a more engaging and social approach, seeing the individual in the context of their environment and a complex socio-economic system (8). This model of communication, focusing on the political, social, family, and structural issues affecting health, has become the preferred model of many communication programmes (9, 10).

**Fig. 1:**
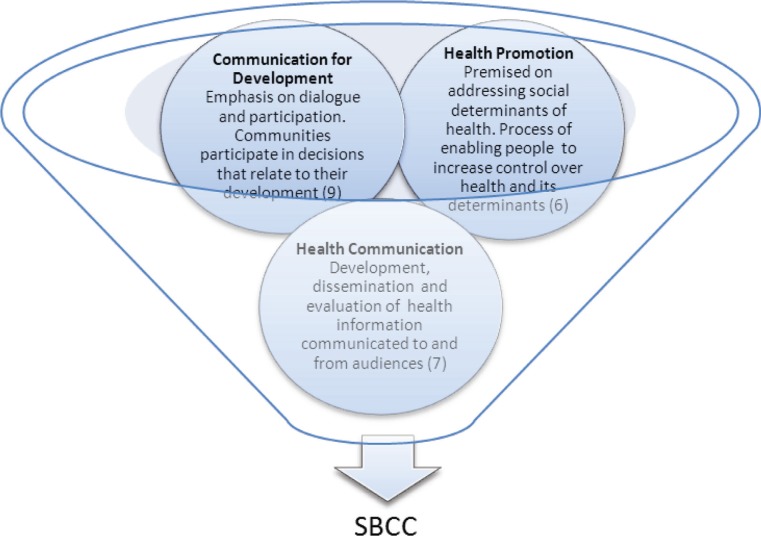
Disciplines influencing social and behaviour change communication.

SBCC is premised on the assumption that individual behaviours need to be understood and tackled within an ecological framework, from interpersonal relations to the policies, cultural norms, and values that shape the world in which individuals live. This requires interdisciplinary collaborations to inform interventions and demands greater engagement with policy making and the political and economic context in the African region (11). In the context of such complexity, SBCC must thus draw from those disciplines that engage in this space, including anthropology, sociology, political science, communication, and marketing, to name a few. To make an impact at the population-level disciplines fundamental to public health such as epidemiology, health policy analysis and health economics make their contribution to SBCC. For example, recently gazette legislation in South Africa addressed the salt content of some processed food, including bread (12). The rationale is that if non-discretionary salt levels can be reduced at the stage of production (creating an enabling environment for decreasing individual salt intake), the country will effectively reduce the salt intake of individuals at risk of developing non-communicable diseases, such as hypertension. This strategy, central to the health promotion concept of making the healthy choices the easier choice, is more cost-effective than attempting to change individuals’ eating habits (13).

SBCC, health promotion, and health communication are not without critics. Governments and donors alike are calling for more evidence-informed health communication interventions. The most common critiques of SBCC tend to revolve around limited evidence of health impact (14–18). To address this communication, practitioners have been arguing that evaluation of SBCC interventions requires alternative methods and greater complexity than single-level or clinical interventions (19). SBCC practitioners have promoted alternative evaluation methods, highlighting that using randomized controlled trials (RCTs) to evaluate multi-level communication interventions is often both unfeasible and unethical (20). This argument may be gaining traction. Recently, some donor agencies have put out calls for alternative ways of evaluating communication interventions. Funding has also been earmarked for continued investment in capacity strengthening for health communication (21).

Thus, SBCC has two challenges to deal with: improving the understanding of interventions to influence population-level changes in behaviour; and developing methods of evaluation to demonstrate the contribution of SBCC to health outcomes and impact. An improvement in the skills of SBCC practitioners in both these areas is required.

SBCC training also needs to account for a great diversity of practitioners, who are employed in a range of institutions, from non-governmental organisations (NGOs) to government health promotion divisions (22). While originating from a range of disciplines, such as marketing, journalism, psychology, education, nursing, medical, or the social sciences, they are all often expected to design, manage, monitor, and evaluate communication initiatives in their workplaces.

The need to strengthen health promotion and SBCC capacity in the global south has been well documented (23, 24). The impact of short-term training or sending a select few individuals to be educated in northern institutions is questionable, as such efforts rarely address the fundamental institutional problems and human retention challenges that underlie capacity gaps in Africa (22, 25–27). The relevance of course content to the challenges faced in the global south has also been under scrutiny. There is growing recognition that capacity strengthening must take place at multiple levels (individual, organisational, sectoral, and institutional), which has led governments and NGOs to explore how universities in the global south can work more closely with networks of other universities, civil society groups, and government to address capacity gaps in the field (22, 25, 28).

The external interest in universities playing a role in SBCC training has been complemented by a university-driven shift to competency-based education (CBE) models (29–31). In the United States of America and Europe, health promotion is an integral part of public health competency frameworks (30, 32). The shift to CBE is a response to a perceived gap between academic training and the practical knowledge, skills, and values required in public health practice, including health communication. Universities have recognised that graduates need to be able to apply their academic skills. This has set the stage for an unprecedented openness to new ways of collaboration for SBCC capacity strengthening within the field of public health.

Defining health communication or SBCC competencies occurred through a parallel process, driven by communication practitioners (4) where academics, government, and civil society actors met in Ica, Peru, in 2001 to develop a competency framework for health communication (25). Health communication, as framed in Ica, is synonymous with SBCC as described earlier. Obregon and Waisbord describe collaboration with higher education institutions as

a critical dimension of sustained and long-term efforts aimed at creating a critical mass of individuals and organizations with a particular focus on the required competencies in health communication. (p. 560) (25)

Some advantages universities offer over NGO training include accreditation (enabling professional growth), standardisation of competencies, structured longer-term programmes, and a higher likelihood of SBCC training being institutionalised and sustained beyond a particular donor funding stream (25).

While university programmes should not be developed in a vacuum, graduates need to return to work environments that allow them to apply their newly acquired skills or else there is a risk that

capacity activities might unintentionally contribute to promoting a sense of frustration among participants who work in organizations that provide little support to change job performance (22).

Successful academic–practitioner collaborations require several elements to be satisfied. These include institutional cultures supportive of evidence-informed communication, establishing trust within the collaboration, and ensuring that the institutions ultimately responsible for training and employing practitioners (versus donors) own the process (22).

The following case study describes and reflects on a partnership between a southern university, the University of the Witwatersrand (Wits), and Soul City Institute, an NGO, to establish an academic, competency-based SBCC programme serving Southern Africa. While northern donors and collaborators were engaged in the process, this case is notable as a partnership between African institutions, leading a capacity strengthening process in Africa to benefit Africans.

## Case study

### Stakeholders

The Soul City Institute for Health and Development Communication, established in 1992, is a large NGO that develops health and development communication and supports a network of regional partners around Africa. The regional support included capacity strengthening, and by 2008, the Soul City Institute had conducted approximately 100 formal training events in areas such as formative audience research, advocacy, print, radio, and television edutainment drama (33). In 2009, Soul City Institute and their regional partners were also experiencing increasing donor demands to demonstrate the health impact of their multi-media and multi-level SBCC interventions as well as staff-driven demand for accredited training opportunities.

The Wits School of Public Health (WSPH) has a long history of responding to the context in which it is located. Historically the academic attention of the WSPH focused on exploring the impact of apartheid on health, research to inform policy to improve health equity in a democratic dispensation, and piloting interventions to achieve improved health system performance. This required that academics worked with civil society, community organisations, and NGOs. More recently, its training programmes have successfully sought to fill capacity gaps in public and population health in Africa (25, 26, 34). WSPH comprises staff drawn from a range of disciplines relevant to public health. Interdisciplinary research and innovative teaching approaches and methods are WSPH standard practice.

### Inception phase of the WSPH–Soul City Institute SBCC training programme

Over and above the development in competency-based approaches to education and the development of the SBCC field described above, three processes informed the development of a SBCC training programme located at the WSPH: an audit of the needs of potential students; a donor commissioned review of SBCC in the region; and later, a baseline assessment of the expectations and experiences of the first intake of students to ensure that our planned curriculum was appropriate.

The audit developed out of a ‘bottom-up’ appreciation of the need for systematic and accredited training at both an individual and organisational level that Soul City Institute garnered through their partnerships with NGOs in Southern Africa. Academic training was identified as a method to enhance professional growth and to enable organisations to implement programmes more effectively. In late 2008, the Soul City Institute conducted an audit with its staff and partners in its regional capacity strengthening programme to explore the educational backgrounds of staff and determine training priorities. The assessment found that most practitioners relied on short-term training to supplement their ‘on the job’ learning. The Soul City Institute had led some of this training. However, as highlighted by Obregon & Waisbord, they encountered challenges with sustaining longer-term training and were unable to satisfy staff requests for accredited training (25).

There was substantial debate at the time about where a sustainable accredited training programme in the field of health communication in Southern Africa could be housed, and whether there was institutional capacity to create long-term in-country health communication expertise in this region. In response to this debate, donors commissioned a review to explore the rationale for locating training within a school of public health as opposed to other options (35). This review began with a search of universities with existing SBCC curricula in South Africa and the Southern African region. A number of opportunities to study health communication or health promotion were identified. However, documentation available about the content showed that none of them entirely represented the interdisciplinary training that SBCC requires. The review identified that practitioners needed to understand the theories and approaches of communication for behavioural and social change. They also needed research and operational skills to plan, implement, monitor, and evaluate health communication programmes at regional, national, district, and community levels (35). The review highlighted that SBCC practitioners needed to understand the epidemiology of communicable and non-communicable diseases and other public health issues, as well as the social, cultural, political, and economic contexts of public health in Southern Africa and thus identified ‘compelling reasons’ for locating the initiative at a school of public health. This included noting that knowledge of health issues was considered vital in order to be able to develop specific and accurate health communication and that there would be a greater opportunity for health communication to be mainstreamed if the programme were integrated into a Master of Public Health (MPH) (35). There were also international precedents for health communication to be located within a school of public health (35).

Baseline research carried out with a sample of SBCC masters’ students confirmed that participants expected to gain a broader understanding of public health, the key principles and meaning of public health beyond medical and epidemiological topics, how to apply SBCC theories in their work, how to implement programmes, and how to measure the outcomes. Almost all participants emphasised the desire to develop skills to monitor and evaluate SBCC programmes. This was identified as the most challenging area in their work. The following quote illustrates the common expectations.

I was expecting to get deeper knowledge on SBCC concepts, theories and approaches for application in my work, including skills on how to design and implement SBCC interventions. I was particularly expecting to learn how to monitor and evaluate SBCC interventions, e.g. media. (MPH student, 2010 cohort)

### The partnership

On the basis of the audit, and supported by the findings of the donor review, Soul City Institute approached WSPH with the idea of a partnership. Both organisations had a long-standing research and training relationship meeting the requirement of trust as highlighted by Obregon & Waisbord (25). This partnership brought together practitioners and a tertiary education institution to develop a master's programme and short courses based on praxis and created a competency-based curriculum.

To take the academic–practitioner partnership further, a consultative meeting was held in May 2009 at the WSPH, with the participation of 16 academics and SBCC practitioners from 10 institutions and five countries to develop the programme. It drew on the dual historical processes of defining competencies both within public health and SBCC and the partnership-defined SBCC competencies relevant to the African context (25, 30). These competencies were then translated into a set of learning outcomes, which became the basis for the curriculum, including SBCC-specific formative research, monitoring, evaluation, strategic approaches, behavioural and social theory, and designing and planning programmes.

The final method of securing the partnership between the academy and practitioners was the establishment of an advisory board made up of practitioners from African institutions and academics from the global north and south. Their brief is to keep the curriculum current and to provide a space for critical reflection. The advisory board reflects the interdisciplinary nature of SBCC and includes SBCC practitioners and academics with a background in communication for development as well as in public health.

### The curriculum

In response to the immediate short-term need for capacity strengthening in the African region, self-standing certificated short courses as well as a field of study in SBCC within the MPH programme were developed. Currently, certificate courses entail a 5-day face-to-face component and some self-study. The courses utilise adult-education pedagogy, which includes space for participants to reflect on their practice and promotes dialogue and discussion, and application exercises rather than didactic teaching Table 1 summarises the SBCC courses developed since its inception.

**Table 1:** Social and behaviour change communication (SBCC) courses

Course name	Year of development	Year first run	Method of delivery
Introduction to SBCC	2009	2009	Short course only
Introduction to health promotion	2009	2010	MPH (2010) and short course
Applying social and behaviour change theory to practice	2009	2010	MPH and short course
Planning and implementing SBCC	2009	2010	MPH and short course
SBCC approaches	2009	2010	MPH and short course
Communication, media and society^a^	2009	2009	MPH and short course
Research, monitoring and evaluation for SBCC	2009	2010	MPH and short course
Entertainment education	2011	2011	Short course only

a Developed and initially implemented by staff from the Ohio University and University of Roskilde.

To illustrate our approach, we describe how we met the specific need for research and evaluation skills through a course on research, monitoring, and evaluation of SBCC. The course addresses formative research principles and methods; pre-testing of draft material, scripts, and approaches; monitoring of SBCC programmes, including the development and measurement of indicators; and evaluation. The evaluation component grapples with the challenges of using experimental study designs in the context of complex SBCC programmes, especially those that use advocacy or community mobilisation approaches. Students are challenged to consider the strengths and limitations of quasi-experimental, mixed method designs and how some analytic approaches such as propensity score analysis can overcome some of the limitations of non-experimental designs. Students conduct their own research during the course which enhances their skills and allows for real-time feedback through a small research project. This course typifies the approach taken in other SBCC courses that form part of the MPH in SBCC.

The MPH in SBCC is systematically evaluated through extensive internal and standardised university evaluation mechanisms. Early feedback from students suggested that the pedagogical approach was meeting their needs.

… my hopes and expectations have changed greatly, largely because what I have learnt is beyond what I had expected and hoped for. For example, the course had afforded skills to measure situations (trends, prevalence, etc.) before intervening. Previously, I took this as sole responsibility of a researcher and not a program manager. I am now able to advise planners and fellow technical people on areas to assess for evidence first before jumping in with interventions. These are skills I did not have before. (MPH in SBCC student at the end of Year 1).

### Demand for SBCC training

The MPH in SBCC was advertised, primarily through Soul City Institute networks and the Communication Initiative website in 2009 (36). Despite this passive form of recruitment, Wits SPH received over 243 applications for the 2010, 2012, and 2013 intakes.

The educational backgrounds, positions, and geographic representation of the MPH in SBCC students indicate the widespread demand for professional SBCC training. The 14 selected for 2010 represented six countries: South Africa, Malawi, Botswana, Zimbabwe, Ethiopia, and Swaziland. They came from diverse educational backgrounds and held positions in NGOs and government (including national AIDS committees) and worked for donor agencies as well as research institutes. Most of the students enrolled for part-time study and maintained their full-time positions as SBCC practitioners. Many were involved in HIV prevention and management. They stated early on that they were particularly attracted by the location of the specialist field of SBCC within the MPH. The practitioners recognised the interdisciplinary nature of the sector in which they worked. Receiving broad-based training in public health enabled the students to engage more confidently with other public health practitioners who played a critical role in the HIV and AIDS sector.

The short course participants had a similar educational profile to the MPH in SBCC students. However, we were able to accommodate some practitioners with substantial SBCC experience who had less than the required formal tertiary education.^1^ The 150 participants who attended one or more of the 14 short courses that ran between December 2009 and March 2012 came from 25 countries mostly in Africa. Interest in attending courses was also received from practitioners in South East Asia including Pakistan and India, the Caribbean, Europe, and the United States of America.

Having a critical mass of staff trained from one organisation can shift the organisational practice. The short courses enabled institutions to send many staff members, often to multiple courses. This has contributed towards building institutional capacity, a need identified in the audit. Some institutions have requested stand-alone or tailored training. Several courses have been run to meet the needs of an international organisation and a UN agency. While the course learning outcomes, structure, and mode of delivery were similar to those routinely delivered, the content was tailored to the needs of the institutions. For example, an agency may request that case studies and in-class exercises focus on reproductive health issues or gender-based violence. Some of this demand has been externally driven or supported; for example, donors have set aside funding for particular types of staff training in SBCC.

Other universities have also demonstrated interest in SBCC capacity development. WSPH has, on request, hosted study tours by universities from Albania in 2010, Tanzania in 2011, and Nigeria in 2012.

### Funding

The SBCC programme received support from a number of donors, including the UK Department of International Development (DFID), the Centers for Disease Control (CDC), and C-Change, which was a 5-year initiative (2007–2012) supported by the US Agency for International Development (USAID) that aimed to improve the effectiveness and sustainability of communication as a component of development efforts. The South African Government's Department of Health contributed through scholarships for their staff. In addition, both WSPH and Soul City Institute invested substantial resources in this initiative. The external support was invested in developing the competency-based curricula and bursaries to enable students who would otherwise not have been able to enrol. This was especially the case for some of the students who travelled from southern African countries. Sustained funding will facilitate the consolidation of the progress made so far to strengthen SBCC capacity in the region. As was evident in the development of a master's programme in epidemiology and biostatistics at WSPH, initial funding has the potential to leverage additional funding and posts from the university and government (34). This is key because building institutional capacity to provide long-term sustainable training requires sustained investments, and once-off (even though multi-year funding) is only paying lip service to stated intentions to build institutional capacity.

## Conclusion

The need and demand for SBCC capacity strengthening in the global south and beyond is clear. Some of this demand is driven from the bottom-up, while some is donor driven. The bottom-up demand has emerged from individuals and organisations wanting to deliver evidence-based programming and to evaluate their interventions systematically. Donors often are motivated by similar goals, especially in an environment characterised by resource constraints. They too want to ensure that SBCC programmes impact on key health indicators. In addition, there is growing recognition that relying on technical support from the global north is not sustainable and that southern-based institutions have the ability and need to be leading capacity strengthening of southern-based organisations and governments. This is especially true for SBCC, where an understanding of the social context and local environment is critical.

The interest that has been expressed by other academic institutions in the global south underlines the understanding that there is demand for home-grown expertise in SBCC beyond the United States of America and northern Europe, where this kind of training has been offered for several decades (25). Collaboration between southern-based academic institutions will further enhance south–south capacity strengthening. The interest from southern-based academic institutions has emerged from a range of disciplines, including communication and media studies, social work, and public health. Engaging across disciplines in this way can further enhance the interdisciplinary nature of SBCC. It also enhances the interdisciplinary nature of public health at WSPH, by exposing MPH students in all fields of study and faculty to students who come from a range of disciplinary backgrounds.

Additionally, the involvement of practitioners and civil society institutions in the conceptualisation, delivery, and review of SBCC course content, as has been the case in the WSPH and Soul City Institute partnership, has reduced the gap between academic training and practical relevance. This partnership has been facilitated by donor support. The degree to which donors will have the flexibility to invest for a sufficient period of time to bed down the institutional capacity now developed in southern institutions is unclear. Continued funding to this partnership and, more importantly, the provision of bursaries for practitioners to attend the courses is uncertain. The extent to which funders will follow suit and invest in southern led intuitions also remains a question.

The degree to which the SBCC programme is actually meeting enhanced capacity at an institutional level is yet to be evaluated systematically. The structure of the WSPH programme is ideal for comparing the effects of once-off short courses to a more integrated set of short courses and the MPH degree. Similarly, the programme structure presents opportunities to explore the impact of individual training to that of institutional strengthening as well as the synergies between the two.

## Footnotes

1 The Health Sciences Faculty, which is the academic home of the Wits SPH, sets minimum educational requirements for entry into the master's programme which includes a 4-year degree.

## References

[1] Gupta V, Goel A (2009). Male circumcision for prevention of HIV transmission. Lancet.

[2] Hallett TB, Singh K, Smith JA, White RG, Abu-Raddad LJ, Garnett GP (2008). Understanding the impact of male circumcision interventions on the spread of HIV in southern Africa. PLoS ONE.

[3] Rose G (1985). Sick individuals and sick populations. Int J Epidemiol.

[4] WHO (1986). The Ottawa charter for health promotion.

[5] WHO (2005). The Bangkok charter for health promotion in a globalized world.

[6] Bernhardt JM (2004). Communication at the core of effective public health. Am J Public Health.

[7] Inter-agency (2011). Communication for development: strengthening the effectiveness of the United Nations.

[8] The Rockefeller Foundation (1997). Communications and Social Change: forging strategies for the 21st century.

[9] de Wit JBF, Aggleton P, Myers T, Crewe M (2011). The rapidly changing paradigm of HIV prevention: time to strengthen social and behavioural approaches. [Editorial]. Health Educ Res.

[10] Soul City Institute for Health and Development Communication (2009). Soul City's theory of social and behaviour change.

[11] de-Graft Aikins A, Unwin N, Agyemang C, Allotey P, Campbell C, Arhinful D (2010). Tackling Africa's chronic disease burden: from the local to the global. Global health.

[12] Department of Health (2012). Foodstuffs, cosmetics and disinfectants (54/1972): regulations relating to the reduction of sodium in certain foodstuffs and related matters. Government Gazette.

[13] Bertram MY, Steyn K, Wentzel-Vijoen E, Tollman S, Hofman KJ (2012). Reducing the sodium content of high-salt foods: effect on cardiovascular disease in South Africa. S Afr Med J.

[14] Noar SM, Palmgreen P, Chabot M, Dobransky N, Zimmerman RS (2009). A 10-year systematic review of HIV/AIDS mass communication campaigns: have we made progress?. J Health Commun.

[15] Ross DA (2010). Behavioural interventions to reduce HIV risk: what works? [Editorial]. AIDS.

[16] Michielsen K, Chersich MF, Luchters S, De Koker P, Van Rossem R, Temmerman M (2010). Effectiveness of HIV prevention for youth in sub-Saharan Africa: systematic review and meta-analysis of randomized and nonrandomized trials. [Miscellaneous Article]. AIDS.

[17] Bertrand JT, Anhang R (2006). The effectiveness of mass media in changing HIV/AIDS-related behaviour among young people in developing countries. [Review]. World Health Organ Tech Rep Ser.

[18] Myhre SL, Flora JA (2000). HIV/AIDS communication campaigns: progress and prospects. [Review]. J Health Commun.

[19] Coffman J (2003). Lessons in evaluating communications campaigns: five case studies.

[20] Do MP, Kincaid DL (2006). Impact of an entertainment–education television drama on health knowledge and behavior in Bangladesh: an application of propensity score matching. J Health Commun.

[21] USAID (2012). Health Comm-capacity RFA.

[22] Waisbord S (2006). When training is insufficient: reflections on capacity development in health promotion in Peru. Health Promot Int.

[23] Atkinson S, Cohn A, Ducci ME, Gideon J (2005). Implementation of promotion and prevention activities in decentralized health systems: comparative case studies from Chile and Brazil. Health Promot Int.

[24] Tang K-C (2005). Building capacity for health promotion–a case study from China. Health Promot Int.

[25] Obregon R, Waisbord S, Obregon R, Waisbord S (2012). Capacity building (and Strengthening) in health communication: the missing link. The handbook of global health communication.

[26] Ezeh AC, Izugbara CO, Kabiru CW, Fonn S, Kahn K, Manderson L (2010). Building capacity for public and population health research in Africa: the consortium for advanced research training in Africa (CARTA) model. Glob Health Action.

[27] Wills J, Rudolph M (2010). Health promotion capacity building in South Africa. Glob Health Promot.

[28] Van den Broucke S, Jooste H, Tlali M, Moodley V, Van Zyl G, Nyamwaya D (2010). Strengthening the capacity for health promotion in South Africa through international collaboration. Glob Health Promot.

[29] Calhoun JG, Spencer HC, Buekens P (2011). Competencies for global heath graduate education. Infect Dis Clin North Am.

[30] Calhoun JG, Ramiah K, Weist EM, Shortell SM (2008). Development of a core competency model for the master of public health degree. Am J Publ Health.

[31] Hagopian A, Spigner C, Gorstein JL, Mercer MA, Pfeiffer J, Frey S (2008). Developing competencies for a graduate school curriculum in international health. Publ Health Rep.

[32] Whittaker PJ, Pegorie M, Read D, Birt CA, Foldspang A (2010). Do academic competencies relate to ‘real life’ public health practice? A report from two exploratory workshops. Eur J Publ Health.

[33] UKAID (2012). Southern Africa regional behaviour change communication programme (DFID MIS: 062-555-01): annual progress report for the period September 2011–February 2012.

[34] Kellerman R, Klipstein-Grobusch K, Weiner R, Wayling S, Fonn S (2012). Investing in African research training institutions creates sustainable capacity for Africa: the case of the University of the Witwatersrand School of Public Health masters programme in epidemiology and biostatistics. Health Res Policy Syst.

[35] Flett P, Haruna E, van Graan M (2008). Review of the establishment of a health communication centre of excellence.

[36] The Communication Initiative Network http://www.comminit.com.

